# Mechanisms of brain self-regulation: psychological factors, mechanistic models and neural substrates

**DOI:** 10.1098/rstb.2023.0093

**Published:** 2024-10-21

**Authors:** Ranganatha Sitaram, Andrea Sanchez-Corzo, Gabriela Vargas, Aurelio Cortese, Wael El-Deredy, Andrew Jackson, Eberhard Fetz

**Affiliations:** ^1^ Multimodal Functional Brain Imaging and Neurorehabilitation Hub, Diagnostic Imaging Department, Saint Jude Children’s Research Hospital, 262 Danny Thomas Place Memphis, TN 38105, USA; ^2^ Institute of Biological and Medical Engineering, Pontificia Universidad Católica de Chile, Diagonal Paraguay 362, Santiago de Chile 8330074, Chile; ^3^ Department of Decoded Neurofeedback, ATR Computational Neuroscience Laboratories, Kyoto 619-0288, Japan; ^4^ Brain Dynamics Lab, Universidad de Valparaíso, Valparaiso, Chile; ^5^ ValgrAI: Valencian Graduate School and Research Network of Artificial Intelligence – University of Valencia, Spain, Spain; ^6^ Biosciences Institute, Newcastle University, Newcastle NE2 4HH, UK; ^7^ Department of Physiology and Biophysics, Washington National Primate Research Center, University of Washington, Seattle, WA, USA

**Keywords:** neurofeedback, brain–computer interface, brain–machine interface, active inference, reinforcement learning

## Abstract

While neurofeedback represents a promising tool for neuroscience and a brain self-regulation approach to psychological rehabilitation, the field faces several problems and challenges. Current research has shown great variability and even failure among human participants in learning to self-regulate target features of brain activity with neurofeedback. A better understanding of cognitive mechanisms, psychological factors and neural substrates underlying self-regulation might help improve neurofeedback’s scientific and clinical practices. This article reviews the current understanding of the neural mechanisms of brain self-regulation by drawing on findings from human and animal studies in neurofeedback, brain–computer/machine interfaces and neuroprosthetics. In this article, we look closer at the following topics: cognitive processes and psychophysiological factors affecting self-regulation, theoretical models and neural substrates underlying self-regulation, and finally, we provide an outlook on the outstanding gaps in knowledge and technical challenges.

This article is part of the theme issue ‘Neurofeedback: new territories and neurocognitive mechanisms of endogenous neuromodulation’.

## 1. Introduction

Neurofeedback (NF) is an experimental technique for closed-loop brain training for animals and human participants to self-manipulate their brain signals [1–7]. NF provides an explicit sensory indicator of a neurophysiological process to enable individuals to modulate activation levels in specific ways to observe its effect on brain function and behaviour. NF has been recognized as a powerful approach to understanding brain–behaviour relationships and has shown potential in basic neuroscientific investigations and clinical rehabilitation to restore and enhance brain function and alleviate cognitive deficits in patient populations [6–10]. Methodological advances and experimental successes of NF led to the rapid development of the brain–computer interfaces (BCIs) and brain–machine interfaces (BMIs), which are methods for enabling humans or animals to directly control external devices without the involvement of peripheral limbs through the learned control of specific features of neural activity [1–3]. The name BCI is generally attributed to non-invasive approaches, based on electroencephalography (EEG), functional magnetic resonance imaging (fMRI) or functional near-infrared spectroscopy (fNIRS). The term BMI is applied to invasive systems developed with electrocorticography (ECoG) and implantable electrodes. Hence, despite differences in implementation technology, methodological nuances and end users, NF, BCIs and BMIs have common neuropsychological principles. Therefore, it is instructive to draw on literature from these approaches for understanding the mechanistic underpinnings of brain self-regulation.

While NF presents promise for clinical treatment, the field still faces several problems and challenges. Studies have shown a great deal of variability among participants in learning brain self-regulation with NF, and many participants fail to learn self-regulation [6–11]. Hypothesized changes in behaviour or symptom improvement do not always follow changes in brain activity due to NF training. Furthermore, the observed behavioural changes or disease improvements typically do not persist over time. The research community often attributes the above problems to the prevalent short training regimens of NF, and calls for more controlled studies and clinical trials, more specific instructions to the participants for carrying out mental imagery, better feedback and reward contingencies, and more sophisticated multimodal and computational tools and techniques [12,13]. Researchers recognize that the neural mechanisms underlying NF learning are only beginning to be understood. NF researchers have expressed the need to develop a theoretical understanding of NF [5,6,14].

The field currently contends with several open questions that could be answered by a better understanding of the mechanistic nature of brain self-regulation. Some of those questions are: What is the optimal NF protocol for learning, and how does it vary with the target brain region(s)? When should mental strategies be suggested and explicitly provided, and when not? What are the optimal ways of providing feedback and reward? How do feedback and reward affect motivation, attention and learning?

It is now well recognized that the lack of sufficient scientific understanding of brain self-regulation adds to the difficulty in overcoming the above problems. Greater insight into the mechanisms underlying NF will help the research community in interpreting the results of data analysis of NF studies, providing explanations for negative findings, designing new protocols, improving existing protocols, controlling the quality of experimental protocols and brain and behavioural data, managing risk to the participants and the study and generating new hypotheses [15].

The purpose of the present article is to review the extant understanding of the neural mechanisms of brain self-regulation by drawing on findings from studies focusing on NF, BCI/BMI and neuroprosthetics. In the following sections, we will delve into the cognitive processes and psychophysiological factors affecting self-regulation, theoretical models of self-regulation, neural substrates underlying self-regulation, and finally, provide an outlook on the outstanding gaps in knowledge and technical challenges.

## 2. Cognitive processes and psychological factors of brain self-regulation

**Figure 1.**
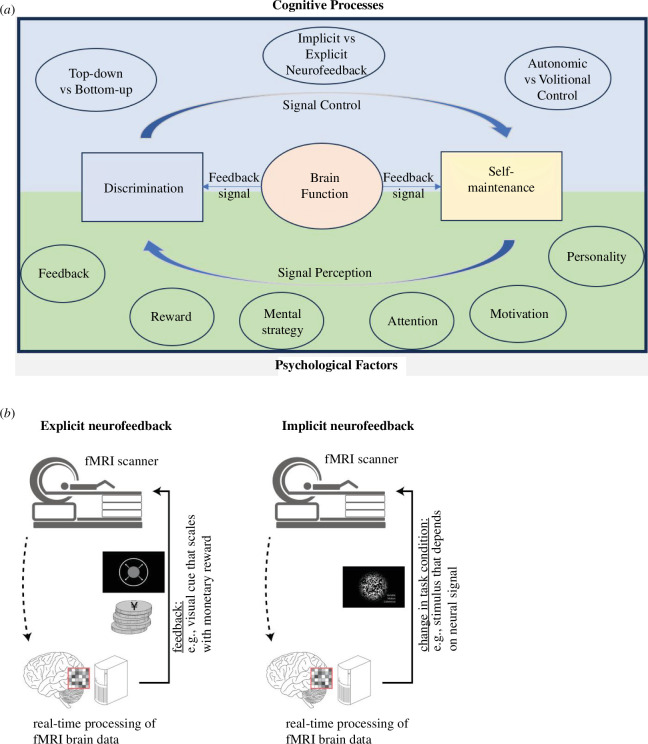
(*a*) Cognitive processes and psychological factors involved in brain self-regulation. Self-regulation of brain activity through neurofeedback (NF) has been proposed to involve two distinct cognitive processes: discrimination and self-maintenance [16]. Discrimination is the ability to perceive and identify the neural variable associated with the feedback signal. Self-maintenance is the ability to affect/influence the neural variable and change it in the intended manner. The hypothesis is that the above two skills acquired through NF training will allow the participant to regulate the neural variable through a volitional psychosomatic process [18]. Cognitive processes and psychological factors affecting NF have been identified in the literature. The most essential cognitive processes are related to top-down and bottom-up processing, implicit and explicit NF paradigms, and autonomic and volitional control. Psychological factors related to NF include feedback type, reward and mental strategy; participant attention and motivation levels; and personality-related features. (*b*) Explicit versus implicit NF. Human NF studies have used two distinct experimental paradigms, ‘explicit’ and ‘implicit’ [17]. During explicit NF, the participant observes the feedback signal as a representation of the biological signal that needs to be controlled during training. In this approach, the participants know they are participating in NF training. In the implicit NF paradigm, the participant does not directly perceive nor is aware of the biological signal. The NF signal modifies the experimental condition (for example, changing the visual stimulus) and the participant does not observe the biological signal in any direct form (modified from Renton *et al*. and Cortese *et al.* [19,20]).

Learning to control brain activity in a specific manner is influenced by cognitive processes modulated by psychological factors [16,17] (see figure 1).

### (a) Cognitive processes

Self-regulation of brain activity through NF has been proposed to involve two distinct cognitive processes: discrimination and self-maintenance [16]. Discrimination is the ability to perceive and identify the neural variable associated with the feedback signal. Self-maintenance is the ability to affect/influence the neural variable and change it in the intended manner. The hypothesis is that, when the above two skills are acquired through NF training, they would allow the participant to regulate the neural variable through a volitional psychosomatic process [18]. Hence, NF training should be evaluated by assessing the acquisition of discrimination and self-maintenance as dual skills. Unfortunately, there is a shortage of empirical or theoretical studies to test the above hypothesis.

Human NF studies have used two distinct experimental paradigms, ‘explicit’ and ‘implicit’ [17]. During explicit NF, the participant observes the feedback signal as a representation of the biological signal that needs to be controlled during training. For example, the participant sees the changes in the haemodynamic activity of the posterior parietal cortex as changing bars of a thermometer, or a circle’s size [21]. In this approach, the participant knows he/she is participating in NF training. The user learns and gains control over the relationship between the neurobiological signal and the changing experimental conditions.

In the implicit NF paradigm, the participant may not directly perceive or be aware of the biological signal, or both. The NF signal modifies the experimental condition (for example, changing the visual stimulus), and the participant does not observe the biological signal in any direct form. Thus, the participant is unaware of undergoing NF training and that the brain signal somehow influences the experiment. To cite an example scenario, as part of an experiment, participants can be asked to play a video game whose parameters (level of difficulty, the number of points won, etc.) change according to the brain activity. Implicit feedback does not provide information about the biological signal to the participant but incorporates indirect interactivity with the participant, thereby influencing the control of brain activity [19,22,23].

Some NF approaches seem to fall somewhat in between the two paradigms, i.e. between implicit and explicit NF. Decoded neurofeedback (Decnef, [20]) is one good example, in that participants know an NF experiment is taking place and receive explicit feedback about the targeted brain activity on each trial; however, they do not know what the feedback and/or how the feedback is related to their task performance [20]. A recent review has clarified the debate by discussing NF in terms of overt and covert processes [24].

There is no precise equivalent of the explicit/implicit distinction for animal NF paradigms, but studies vary in the extent to which animals are provided with feedback signals. Early animal NF studies delivered a reward, often associated with sensory cues, to train subjects to modulate brain signals. For example, the deflection of a meter arm could provide monkeys with a continuous signal showing how close their neural activity was to a reward threshold [25]. The meter deflection could signal complex combinations of neural and muscular activity, and as these combinations were changed, monkeys quickly changed their response patterns as required to obtain a reward [26]. Such NF could also signal neural patterns unlikely to have motor or sensory correlates, such as the relative number of epileptic bursts and normal firing of single cells in an epileptic focus [27].

More recently, the development of BMI paradigms prompted a renewed interest in NF. BMI-NF paradigms using non-human primates typically begin with subjects controlling a visual cursor with natural movements (e.g. reaching with the arm) while the activity of a large number of neurons is recorded [28]. This provides an initial training dataset to build a decoder that allows the cursor to be controlled directly by neural activity. In some experiments, this decoder has then been perturbed systematically, for example, by changing how some or all channels influence cursor movement requiring animals to learn a new control strategy for the BMI-NF [29,30]. Evidence suggests that the neural activity associated with natural movements may bias the exploration of these new strategies. One method to analyse the population activity is to represent the instantaneous state of all the recorded neurons as a point in a high-dimensional neural ‘state-space’, where each dimension reflects the firing rate of a single neuron. Because the activity of individual neurons is often correlated with others, it is possible to extract lower dimensional subspaces or ‘neural manifolds’ within the full state-space that are associated with the movements in the training dataset. Evidence suggests that if BMI-NF control can be achieved with activity patterns that remain within these manifolds, it can be learned more quickly than a BMI-NF task that requires activity outside of the manifold [30]. One interpretation of this finding is that BMI-NF learning, at least within a single session, consists of a rapid reassociation between a pre-existing repertoire of activity patterns and the new goals of BMI-NF control [31]. Note that the subjects in these experiments typically would have been extensively trained with natural movement tasks prior to any BMI-NF protocols being introduced, and such pre-training may play a role similar to explicit instructions in human NF studies that establish an *a priori* expectation about strategies that might be successful. Alternatively, the neural correlations that constrain the neural manifolds may be imposed by the connectivity within the network of neurons. In any case, acquiring ‘off-manifold’ solutions typically requires several days of training [32]. Such multi-day learning is associated with an initial reduction in correlations between neurons (interpreted as an unstructured, trial-and-error exploration) followed by a progressive increase in correlated variability (interpreted as the formation of a new neural manifold appropriate for the BMI-NF) [33]. Similar learning processes may be required in those human NF tasks where an appropriate mental strategy is not provided in advance to subjects.

NF researchers have conflicting views on whether NF training leads to subconscious regulation of brain activity or represents volitional regulation brought about by the participant’s intention. Some reports suggest that NF is more efficient when based on volitional self-regulation with consciously chosen mental strategies [34], while others maintain that regulation can be achieved without volitional control [7,35,36]. Except for a very few empirical studies [37], little has been done to directly test the above two hypotheses. Sepulveda and colleagues found through an empirical comparison of NF parameters that the feedback signal resulted in a significant learning effect. However, anecdotal reports by practitioners have expressed that feedback learning was hard to achieve until a cognitive strategy (e.g. imagery) was provided. The effectiveness of learning with or without volitional control probably depends on a variety of factors, including context, experimental paradigm, as well as the targeted brain state (e.g. the overall activity in the brain area or the pattern of activity reflecting some specific representation).

Another major question is whether brain self-regulation is a top-down or bottom-up neural mechanism. Top-down processing involves higher cognitive functions originating predominantly in the prefrontal cortices, leading to downstream processing in the sensorimotor and association areas [34,38]. Bottom-up processing originates in the primary and secondary sensory processing areas and drives prefrontal cortical brain activity [39]. These two views may lead to different ways of designing and conducting NF. Based on the bottom-up view, an experimenter may design NF training in terms of conditioning strategies, reinforcement schedules, shaping and chaining [23,40,41]. The top-down model may form the basis for instructing participants to use mental imagery to regulate activation in different brain areas.

To resolve the above contradiction, some researchers have proposed the dual-process mechanism of NF [5,42], according to which both the bottom-up and top-down mechanisms actively interact to support NF learning depending on the task requirements. The bottom-up, operant conditioning-related NF involves automatic and capacity-free processes, while the top-down, cognitively involved NF is a controlled and capacity-limited process. At the connectionist network level, the two processes could be seen as integrated in a network that combines the two separate top-down and bottom-up networks. In the following, we will elaborate on two extant models, namely, reinforcement learning and active inference, which represent two leading models roughly mapping onto the above two opposing approaches. In §3, we will discuss theoretical models of brain self-regulation based on rigorous mathematical formulation that enable researchers to conduct computational simulations and empirical tests to test the above two theories of brain self-regulation.

### (b) Psychological factors

The NF literature suggests that the following experimental conditions and variables may influence NF learning and performance: feedback contingency, reward, instructed or self-developed mental strategies and duration of the training [6]. The major psychological variables in empirical literature include attention, motivation, mood and personality [43].

Contingency refers to the conditional probability of reinforcement, in terms of feedback and reward, given behavioural and neurophysiological responses or a failure to respond. The study of feedback and reward contingencies in the NF literature has been limited [37,44]. Investigation of feedback contingency would include studies on different modalities (visual, auditory, tactile, etc.) of the response-contingent stimuli, their different physical properties (such as amplitude, rate and complexity) and the different functional relationships between the response and the feedback [17].

NF learning is affected by the temporal contiguity of feedback and reward, which refers to the time interval between the neurophysiological response and the presentation of the reinforcement [45,46]. In the context of fMRI and fNIRS NF, the intrinsic delay (in seconds range) between the neural activity that is being regulated and the blood oxygenation-dependent (BOLD) signal changes due to the slow haemodynamic response is a critical factor to be considered, too. In comparison to the BOLD response, the scalp EEG has excellent temporal resolution with millisecond precision, allowing for a higher signal-to-noise ratio in the feedback information due to more samples of the data per time point [47]. In general, the onset of the haemodynamic response, measured by fMRI [48–50] and fNIRS [51,52], lags neural activity in the seconds range (the BOLD response, typically, starts 1 s after neural activity, takes 6 s to peak and takes 11 s to return to baseline from the onset of the haemodynamic response [53]). However, recent studies have shown that the BOLD response to neuronal activity can be more nonlinear and rapid than is commonly held, suggesting that fMRI can reflect fast neuronal dynamics [54]. The time involved in the acquisition and computation of the feedback signal would add to the delay in the feedback, affecting its temporal contiguity. Whether such delayed temporal contiguity degrades or improves self-regulation learning is an open question [6]. A study showed that intermittent feedback (about 20 s delay) is more effective than continuous feedback presentation when the participant uses mental imagery for self-regulation [44]. The argument explains that intermittent feedback could be more advantageous in certain situations, such as during the early stage of learning because it does not interfere with ongoing mental imagery and associated dual-task cognitive overload. Whether intermittent or continuous feedback is more effective in explicit and implicit NF studies is still to be investigated. Some concrete indications come from simulation work, where cognitive strategies responded better to continuous feedback while intermittent feedback instead was more effective for automatic reward-based learning in implicit NF [35].

In animal studies operantly conditioning firing rates of neurons in the motor cortex, the NF signals can be instantaneous, providing signals that even precede any associated movements, which typically occur approximately 100–200 ms later. Often, feedback is multi-dimensional, like cursor control in two or three dimensions. Therefore, feedback signals can be highly informative in spatial and temporal domains. In this respect, at least, BMI-NF perturbation experiments share similarities with traditional motor learning paradigms. For example, the perturbation used by Jarosiewiczs *et al*. [29], in which the mapping of a subset of neurons to cursor movements was rotated, generated errors like classic visuomotor rotation perturbations (in which the mapping from arm movement to the cursor is rotated) [29]. This may explain why the predominant strategy seen in response was a global re-aiming to rotated targets. Motor learning involves multiple processes occurring over different time scales in various brain areas [55], with a fast error-based learning system probably involving the cerebellum [56]. This rapid recalibration of the sensorimotor map may be equivalent to the early reassociation within the neural manifold in BMI-NF paradigms. By contrast, a slower-to-learn but also slower-to-forget reinforcement learning system responsible for skill learning preferentially operates when only low-dimensional ‘success-based’ feedback is available [57], probably dependent on long-term potentiation-like plasticity mechanisms involving the motor cortex [58]. In the context of BMI-NF paradigms, this slower skill-learning system may underlie the emergence of new neural manifolds over several training days. Evidence in rodents suggests that such NF learning may rely on corticostriatal plasticity [59] and can be prevented by optogenetic inhibition of the basal ganglia [60]. Moreover, operant conditioning of neural activity in freely behaving monkeys can be achieved with feedback mediated through stimulation of reward centres alone [61]. By this reasoning, human NF studies, especially those based on haemodynamic signals with significant time delays mediated by low-dimensional ‘success-based’ reinforcement rather than informative error signals, might similarly be expected to depend on corticostriatal skill-learning mechanisms. Interestingly, the consolidation of such long-term skill learning depends on sleep and, in particular, the reactivation in sleep of patterns of neural activity associated with the performance of the skill when awake [62]. This reactivation process involves the repeated firing of neural circuits that were initially used when learning the task, thereby strengthening the synapses that encode the learning. Studies in rodents have similarly shown that neural ensembles involved in BMI-NF task learning are reactivated in subsequent sleep [63]. However, the role of sleep-dependent consolidation processes in human NF studies has been surprisingly neglected.

Two major procedural elements have been used in extant studies for training voluntary self-regulation in humans: instructions and response-contingent stimulation [5,17]. Animal studies of NF and BMIs have strictly relied on feedback and reward following the operant learning paradigms [25]. While experimental work on voluntary control has tended to focus more on the role of feedback, older NF studies observed that instructions are not neutral in influencing voluntary control [64]. Hence, the investigation of experimental instructions is essential for the analysis of voluntary control. Furthermore, more recent developments in BCIs and real-time fMRI NF have emphasized designing experiments incorporating sensitive yet robust measures that control placebo effects and instruction with control conditions [65]. While providing explicit instructions to human participants to use mental imagery and monetary reward is quite common in recent work on NF to enhance and accelerate learning, the optimal strategy for improving volitional control remains unclear.

During the early period of biofeedback development, sophisticated procedures of operant training, such as shaping and chaining, received attention in the literature [66]. Such approaches have the potential to improve learning brain self-regulation. However, more recent work on human NF in EEG and fMRI studies has not explicitly investigated them.

Sepulveda *et al.* [37] investigated the differential effect of feedback, explicit instructions and monetary reward on healthy individuals to up-regulate the BOLD signal in the supplementary motor area (SMA) using fMRI NF. Four groups were trained in a two-day protocol: (i) group F with feedback only, (ii) group FR with feedback and monetary reward, (iii) group FI with feedback and instruction for carrying out motor imagery, and (iv) group FIR with feedback, monetary reward and instruction. Their results showed that group F participants learned to increase the BOLD signal in the SMA significantly from day 1 to day 2, while the other groups did not show a significant learned increase. Additionally, group FR attained the highest BOLD signal amplitude in SMA during the training, although the rise of the SMA signal was not significant from day 1 to 2. The two groups instructed to use motor imagery did not show a significant learning effect over the two days [37]. It is possible that the learning curves may be quite different across manipulations, i.e. a fast-learning curve quickly reaching a plateau for group FR and/or FIR (thus not showing a significant increase from day 1 to 2) as opposed to a slow-learning curve in group F (thus showing a significant increase from day 1 to 2). It is unclear whether these effects are specific to the SMA and the type of mental imagery instructed in the above study. More studies in different functional regions and brain networks are required to consolidate our understanding of the effect of experimental factors on self-regulation.

A major challenge in human NF studies is to find an effective reward as extrinsic motivation for human participants (i.e. food, money, gifts, encouraging words). Extrinsic rewards, however, can often have a negative effect on performance. Lepper *et al*. [67] showed through their psychological experiments that extrinsic reward may act as short-term stimulation but induce longer term depression of motivation [67]. How intrinsic reward should be identified and measured online and reinforcement provided accordingly are still open questions. A working hypothesis is that NF training is expected to develop the above two primary cognitive skills for successful regulation: discrimination and self-maintenance [17]. Accordingly, if NF training leads to improvements in the above two skills, intrinsic motivation would be rewarded. Identifying cognitive strategies and experimental parameters to improve those skills may be more effective as rewarding information than extrinsic rewards such as money or food. Providing instructions to participants for using mental strategies and imagery could lead to enhancing intrinsic motivation.

Recently, Kadoush & Staunton conducted a systematic review of the NF literature to evaluate the psychological factors that influence NF learning carefully [68]. Their findings showed that attentional variables are significant to both performance and education; motivational factors and mood were moderate predictors of success. The authors suggest that future research should systematically manipulate psychological variables such as motivation or mood. Furthermore, non-responders could be tested with different NF parameters to understand whether their non-response is specific or general [68]. Such research will help develop better approaches for non-responders and improve the efficacy of NF.

## 3. Models of brain self-regulation

In this section, we will consider two major models of brain self-regulation, namely, reinforcement learning and active inference, which are two rigorously formalized models for analysis and comparison.

### (a) The reinforcement learning model

Reinforcement learning (RL) is a general computational/modelling framework concerned with how agents learn to select actions that will maximize future cumulative rewards. In its original form, RL was discussed primarily to explain associative learning by which an association between two stimuli or between a behaviour and a stimulus is learned. Associative learning states that the probability of a physiological response is increased when a reinforcing stimulus follows that response. The theory focuses on three parameters, namely, discriminative stimulus, responses and reinforcers. When the response is reinforced in the presence of a discriminative stimulus and no other stimuli, the increase in response probability will occur only in the presence of that stimulus. In the case of an NF experiment, a reinforcing stimulus could be the feedback of the brain activity, for example, in the form of an increase in the bars of a thermometer in proportion to the amplitude of the BOLD signal in a brain region relative to baseline or a given reference activity.

Two different mechanisms of RL have been proposed in the literature [17,69,70]. Model-based RL is goal directed and based on the participant’s internal model, e.g. in explicit NF, the participant has a goal to regulate the feedback signal in a specified direction [71,72]. In the model-free RL, the participant has no model of the environmental events and learning happens by the simple association of stimulus and response, e.g. in implicit NF, the participant does not receive information about the feedback signal directly [71].

Certain critical components of RL, like the choices made by participants regarding behaviour and exploration while learning, the predictions they form about the results of their choices and the ensuing prediction errors, are often overlooked or not recorded in practice and not included in NF analysis. As a result, the significance of these underlying parameters in NF learning has not been fully and systematically explored. It is necessary to recognize the two most critical dimensions in NF that define structurally different learning regimes to establish the correct interaction of the RL elements applied in NF. The first dimension involves the goals of regulation, which are mainly divided into explicit and implicit (although intermediate protocols exist). The second dimension is the feedback presentation time that separates continuous protocols (immediate feedback) from intermittent ones (delayed feedback or separated from the regulation phase).

Lubianiker *et al*. [39] proposed a general framework of NF based on RL to understand and evaluate the parameters that guide the learning of neural regulation. They suggested that this framework can generate new proposals to enhance the effectiveness of NF protocols [39]. In their model of RL, agents learn which actions they should take, guided by environmental reinforcement. In their framework, RL models the environment with three elements: states, actions and rewards. In NF protocols, actions change the state of the environment, and agents learn a policy that maps states to actions to maximize long-term rewards. Rewards depend on states and actions. Lubianiker *et al*. [39] formally show how RL elements, such as value functions, policies and credit assignment problems, are represented in different NF protocols. We present below a summary of the formal elements, but please refer to Lubianiker *et al.* [39] for a more in-depth understanding.

States at a specific trial n and point in time t , i.e. $x_{n}\left(t\right)$ , depend on the previous state $x_{n}\left(t-1\right)$ and the action taken at that time $A_{n}\left(t-1\right)$ , given by

$x_{n}\left(t+1\right)=f\left[x_{n}\left(t\right),A_{n}\left(t\right)\right]+\varepsilon_{n}^{x}(t)$ , where $\varepsilon_{n}^{x}(t)$ is the state noise.

Actions A , understood as internal or external bodily movements, are a fundamental component in RL problems. In NF, actions are purely mental and hence neuronal events. An action at a specific trial n and point in time t , $A_{n}\left(t\right)$ will depend on the overall intentional strategy $A_{n}$ and the subconscious strategy $a_{n}\left(t\right)$ , given by

$A_{n}\left(t\right)=A_{n}+a_{n}\left(t\right)+\varepsilon_{n}^{a}(t)$ , where $\varepsilon_{n}^{a}(t)$ is the action-related noise.

Rewards are the third central element in RL. Rewards can be immediate, associated with a specific action and state, or long term, determining the success of a response. The immediate reward $r_{n}\left(t\right)$ is a function of the state, given by

$r_{n}\left(t\right)=g\left[x_{n}\left(t\right)\right]+\varepsilon_{n}^{r}(t)$ , where $\varepsilon_{n}^{r}(t)$ is the reward noise.

In explicit protocols, actions are usually mental strategies that participants explore, whose space may be limited or expanded according to task instructions. In explicit protocols with intermittent feedback, states are separated for regulation periods $x_{regulate}$ and for reward presentation and evaluation of action utilities $x_{feedback}$ . Intermittent feedback faces the challenge of temporal credit assignment, which is the delay between the neural activity event and the subsequent feedback. Temporal credit assignment involves determining how credit should be assigned to intermediate actions in a sequence of decisions. However, intermediate feedback has the advantage of separating regulation and feedback states [73,74]. On the other hand, explicit protocols with continuous feedback do not have the temporal credit assignment problem because the feedback is provided continuously, but the participant’s attention could be split between conscious action and evaluation of its utility since we have a combined state $x_{regulate+feedback}$ .

A key question is what determines the ‘state’ in RL? Based on previous work on neural representations, and the spectrum of activity patterns that can be elicited at any given time by a population of neurons, it is evident that neural systems are much more constrained than they would superficially appear [31,75]. This means that, even in the context of fMRI signals, the mapping between fMRI multivoxel patterns and the underlying neural activity patterns are hypothesized to be closely matching [7,36].

What evidence do we have that RF is a plausible mechanism underpinning NF learning? Simulation work has shown activity patterns in artificial neural networks can be reinforced via a simple learning rule that updates the weighted connections between units in a manner compatible with standard decoder-based NF experiments, by computing a feedback signal from smoothed and delayed neural activity decoding (akin to fMRI, [36]). Similarly, Oblak *et al*. [76] demonstrated through simulations that intermittent feedback of decoded neural activity can be rapidly learned automatically, i.e. consistent with a basic RL mechanism [76]. A re-analysis of published work, particularly of decoded NF experiments, has highlighted how feedback elicits commensurate activity in the basal ganglia and medial prefrontal cortex [36], areas typically involved in RL processing [77–79]. Interestingly, the orbitofrontal cortex shows activity tracking cumulative failures in NF training [80]. Hence, learning to self-regulate brain activity is consistent with learning goal-directed actions, as in model-based RL. Finally, a recent study found the grey matter volume of the right putamen—a key region in associative/instrumental learning—to be predictive of NF success [81].

Animal literature is richer in studies that have explicitly linked BCI/BMI control of neural activity to RL mechanisms. In a foundational study, Koralek *et al*. [59] demonstrated that learning intentional neuroprosthetic skills depends on corticostriatal circuits and striatal synapses receiving cortical inputs (e.g. striatum and interactions between the striatum and motor cortex). The brain appears to learn faster to control new activity patterns that fall within existing neural population structures, as opposed to entirely new activity patterns, inconsistent with existing neural network structures [30]. The rapid co-optation of a pre-existing structure to achieve new behavioural outcomes resembles model-based RL, whereas the longer term development of de novo neural strategies based on unguided exploration may be better described by model-free RL. An interesting hypothesis postulates that RL operates directly on neural dynamics [82]. RL provides a simple rule to change neural firing rates, which results in altered dynamics. This mechanism can also provide a solution to the issue of how the brain can re-enter a certain state (as a trajectory in neural state space) and refine over time cortical dynamics to achieve target states more reliably [82]. Given that RL seems widely present in neural computations related to learning and behaviour changes (e.g. even visual perceptual learning can be accounted for by RL, see [83]), RL presents itself as a plausible model for explaining NF learning.

### (b) The active inference model

Active inference is a theoretical framework for understanding learning and decision-making in the brain, based on the free energy principle [84–88]. It posits that organisms constantly minimize surprise or uncertainty in their environment by making predictions and actions. This is achieved by the brain constructing internal representations (models) of the environment and generating predictions about incoming sensory information. When the internally generated predictions do not match reality, a prediction error arises, requiring the brain to update its internal models, to improve future predictions, thus reducing future surprise or uncertainty. During perception, the brain anticipates sensory input, allowing it to actively sample information that aligns with its predictions, thus minimizing discrepancies. During action, the brain selects behaviours that minimize the discrepancy between predicted and actual outcomes, leading to adaptive responses. This process drives learning by encouraging the selection of actions that align with desired outcomes. By viewing NF learning as a process of minimizing uncertainty, active inference may contribute to our understanding of how brain self-regulation becomes possible.

Constructing internal models entails inferring the hidden (unobservable) states of the world that cause the observations, and optimizing actions to update the models or to adapt to change. It is suggested that this is carried out by using Bayes’ rule in a variational scheme [86]: the brain generates a probability distribution of the sensory information (*p*) and optimizes it using an approximate distribution (*q*). The optimization borrows the concept of ‘free-energy’ from statistical physics, where free energy represents an upper limit on the difference between (*p*) and the (*q*). Minimizing free energy corresponds to maximizing the brain’s ability to predict and control its environment effectively.

**Figure 2.**
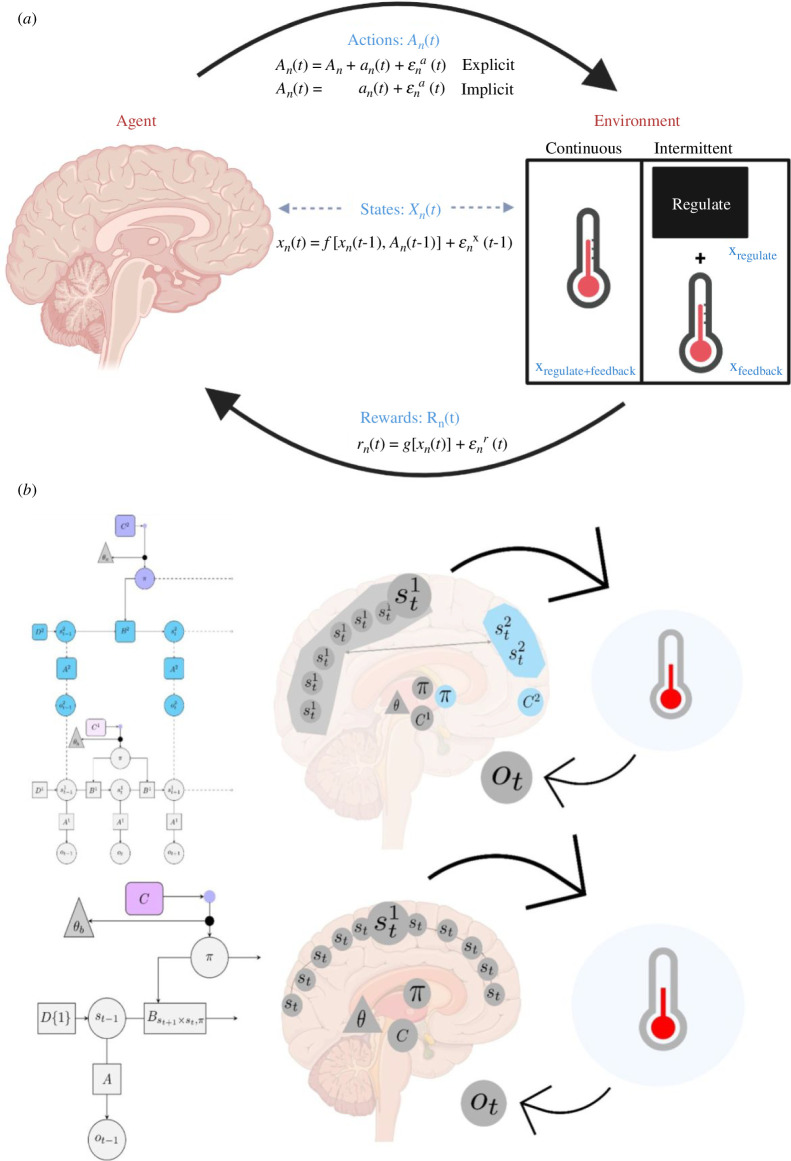
Reinforcement learning (RL) and active inference models. (*a*) RL elements in neurofeedback (NF). In the context of NF, the agent is the NF trainee, and the environment is the task interface that informs the agent about its states, identified as *x_n_* (*t*) at time *t* during trial *n*. These states correspond to changing cues within the task, such as 'rest' or 'regulate', along with observations that are assessed for rewards *r_n_* (*t*). The agent engages in cognitive actions, denoted as *A_n_* (*t*), which impact the transition between different neural states—essentially the brain’s neural dynamics across various regions (also represented as *x_n_* (*t)*). This activity, in turn, modifies the feedback received, creating a feedback loop. This feedback loop blurs the lines between the agent and the environment within NF frameworks, a phenomenon that intertwines their roles. Created in Biorender and adapted from Lubianiker *et al.* [39]. (*b*) Computational neuroanatomy of brain self-regulation by active inference. Computational architectures of active inference-based generative models, within a partially observable Markov decision process (POMDP) framework [89] are depicted, with hierarchical structure (top) and without hierarchical structure (bottom), in the context of an NF task. The mapping of model elements (left column) to anatomical brain areas (right column) aims to match information processing and the corresponding anatomical networks based on existing knowledge and theoretical assumptions regarding brain regions and their interactions [87,90–92]. This highlights the importance of hierarchical architecture in simulating meta-awareness and controlling mental actions, reflecting top-down information processing observed in brain data. The model uses notions of a POMDP [89], encompassing hidden states (*s*), observations (*o*), likelihood matrices (*A*), transition matrices (*B*), expected action sequences or policies (*π*), parameters capturing prior beliefs (*C*), and memory formation (*θ*). Following an NF experiment, the agent infers the hidden brain states (*s*) from NF observations (*o*) and the consequences of mental actions (*π*). Mental actions (*π*) transition brain-hidden states from the current state ‘*s*’ at time step (*t* + 1) to a new state ‘*s*’ according to the transition probability represented by matrix *B*. Sensory observations (*o*) from the new state are generated from a likelihood distribution given by *A*. The search is characterized by a high volume of hidden states and narrow observations (such as a visual thermometer). In the one-layer structure, the model relies solely on simple prior preferences (grey *π*) and connectivity between cortical and subcortical regions (grey *C* and *θ*), serving as controllers for inference and action selection. In contrast, the hierarchical structure adds specialized structures to the planning process (blue *π*, *C* and *s*), such as the observed functional role of frontal areas, enabling more efficient mapping between states, observations and mental actions. Learning is achieved by formulating a strategic plan involving precise policy selection based on anticipated action consequences. The hierarchical architecture effectively demonstrates learning by integrating precise modulation in policy selection, thereby enhancing confidence in cognitive actions and optimizing the decision-making process. In contrast, non-hierarchical structures exhibit unstable and variable action selection without precision, resulting in lower efficiency levels and indicating a lack of strategy.

During NF training, the generative model in the brain quantifies the relation between sensory observations from the feedback (*o*) and the brain’s hidden states that caused them (*s*). This relation is captured by a likelihood mapping (*A*), which encodes beliefs about how the brain’s hidden states are related to the observations that they generate (figure 2*b*). The generative model also accounts for the knowledge of the temporal dynamics of states in the form of beliefs about state transitions. This is captured by a transition matrix (*B*), which encodes the probability of being in some brain state *s* ^1^ at some time step *t* + 1, given that the system was in brain state *s* ^2^ at time step *t*. The control of the transitions is made by the policy (*π*), a belief over the sequence of mental actions (*a*), with *π* = (*a*_1_ *,a*_2_ *,...a_T_*) in the time horizon *T : t*_1_ *,...,t_n_*; where policy selection is implemented through the updating of beliefs about state transitions, informed by the consequences of mental action.

Two parameters influence policy selection: the prior preference (*C*) and the prior policy precision (*γ*). The parameter *C* defines the agent’s pre-existing beliefs about sensory outcomes, irrespective of the policy’s free energy. Essentially, it represents the inclination to anticipate positive feedback and evade negative feedback. On the other hand, *γ* encapsulates the agent’s prior beliefs concerning the level of confidence with which policies can be inferred, contingent upon the policy’s free energy. Policy selection implies choosing the mental action (sequence of transition matrices) most likely to yield the expected outcomes and reduce the uncertainty of hidden states, a process influenced by prior preferences.

Given the novelty of active inference, there is limited literature on applications to NF. Mladenovic *et al*. [93] suggested that integrating active inference into a BCI exhibited significant improvements in performance compared to conventional methods. They observed variation between subjects and noted the potential for tuning the active inference’s parameters to account for each subject’s dynamics.

We constructed a self-regulation model based on active inference (figure 2*b*) to test scenarios about how top-down control could account for the variability in self-regulation learning, and the resolution of uncertainty when individuals seek to optimize their mental actions. Non-learners could be simulated by a model of a single layer guided by the prior probability of achieving an external goal, and connectivity between cortical and subcortical regions serving as controllers for inference and action selection (figure 2*b*, bottom). Landing on the brain state and mental action that explain the observed NF becomes an exhaustive computational task that is achieved by chance. Learners on the other hand are best modelled by a hierarchical structure connecting cortical areas, subcortical regions and frontal areas, allowing a more efficient access to the correct mental actions (figure 2*b*, top).

## 4. Neural substrates of brain self-regulation

One of the earliest studies that attempted to identify the neural correlates of self-regulation was conducted by Hinterberger *et al*. [94] using a slow cortical potential (SCP) BCI simultaneously with fMRI acquisition, showing that learning SCP control correlated with the activation of striatal and motor networks related to the associative binding of behaviour to the reward. Another study investigated the fMRI correlates of EEG-based brain self-regulation and identified the activation of the bilateral anterior insular cortex (AIC), anterior cingulate cortex (ACC), supplementary motor area (SMA), dorsomedial and lateral prefrontal cortex (DLPFC) and the posterior parietal cortex (PPC) [95]. A meta-analysis of fMRI NF studies showed similar results as in the above studies with correlated activity during self-regulation in AIC, ACC, SMA, DLPFC and PFC, PPC, basal ganglia and thalamus [96]. An interpretation of these results [5] attributed the different brain correlates of NF to a reward-processing network (comprising the ACC, AIC and ventral striatum), a control network (comprising the lateral occipital cortex (LOC), DLPFC, PPC and thalamus) and a learning network (involving the dorsal striatum). Animal studies have contributed substantially towards identifying the brain regions responsible for feedback learning, demonstrating, for example, that the complete blockade of NMDA receptors in the basal ganglia abolishes learning [59,97]. The brain’s structural predictors of self-regulation and changes resulting from training have also been investigated (e.g. [98–100]).

**Figure 3.**
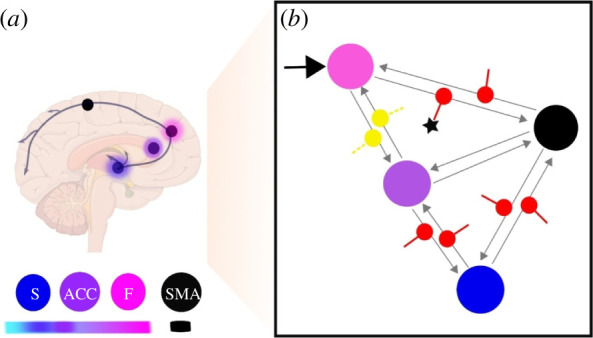
Self-regulation learning as active inference: dynamic causal modelling of an fMRI NF task (reproduced with permission from Vargas *et al*. [38]*).* A schematic illustration of the most probable model and parameter configuration of brain self-regulation learning. (*a*) The same network as (*b*) represented as brain regions, with nodes indicating the different brain regions and their hierarchical identification shown as colour labels following the functional anatomy of hierarchical active inference [90]. Different levels are represented by a colour gradient from fuchsia (frontal lobes, highest level) to blue-cyan (striatum). F, frontal lobe; ACC, anterior cingulate cortex; S, striatum; SMA, supplementary motor area. (*b*) The network connectivity during self-regulation and the significant difference between learners and non-learners. The input connection (*C* matrix) is indicated by a black arrow, while the effective connectivity architecture (*A*e matrix) is represented by grey arrows. The modulation of extrinsic connectivity values (matrix *B*e) is depicted as red for excitatory and yellow for inhibitory. The star symbol denotes significant differences (*p* < 0.05) in connectivity between learners and non-learners. Specifically, it highlights variations in excitatory modulating connectivity between the frontal and target areas; with ‘learners’ corresponding to participants who effectively increase or decrease brain activity through NF training, evidenced by a significant increase in activity within the target region from the beginning to the end of the intervention, while non-learners do not exhibit this change.

A recent work by Vargas and colleagues conducted a direct evaluation of top-down and bottom-up processing of brain self-regulation, in the context of active inference [38] (figure 3). They performed dynamical causal modelling (DCM, [101]) of a real-time functional MRI NF dataset based on the work by Sepulveda *et al*. [37] to investigate the neural architecture that enables self-regulation learning and distinguishes learners from non-learners.

The authors evaluated the DCM models to assess which network structure is more likely to have generated the observed brain self-regulation data [37]. They also tested the difference in the brain networks of participants who successfully learned brain self-regulation and those who did not. According to their results, self-regulation learning was best modelled by a hierarchical architecture based on an active inference framework. In this architecture, the effects of feedback and self-regulation were mediated by top-down connections in the frontal cortex (including frontal superior and medial frontal gyri), anterior cingulate cortex and the striatum, together called the F-ACC-S axis. The results reveal different network modulation weights between NF learners and non-learners. These results could be viewed in the general context of learning, where active inference [90,101] suggests a shift from striatal bottom-up processing to frontal top-down influence as a possible explanation of learning [102].

It should be noted that active inference and RL models are not mutually exclusive. Active inference integrates a hierarchical neural architecture of RL-based learning by trial and error mediated by reward [103] with prior internal information used in generative predictions [102,104]. In this integrated framework, the generative model is updated by bottom-up sensory signals, thus increasing the predictive validity of the model that affects the top-down processes. This mechanism has been considered to result in better predictions and resolution of prediction errors originating in the striatum. The difference in top-down predictions based on frontal prior beliefs differentiates learners and non-learners. Based on this new view, relying only on bottom-up signals during self-regulation learning would be an untenable learning strategy due to the dimensionality of the search space and time constraints, and states that for self-regulation learning to succeed, top-down control may be necessary. This assertion is supported by findings in the cognitive functions that show that the internal generative model [90] is present in learning [105], abstract construction [106] and cognitive control [107].

However, both old and more recent work has shown neural activity in the context of brain self-regulation to be much more constrained than we might expect [31,75]. Simulations of fMRI NF have equally shown that, counterintuitively, the dimensionality of the search space may not be so high, leading to relatively fast learning [7,35,36]. Given that complex behaviours and information processing can be performed without awareness [108–111], there is substantial evidence suggesting we may not need conscious awareness, or explicit cognitive strategies, to exert control over our own neural activity. A probable scenario is that the brain has multiple (hierarchical) mechanisms for self-regulation. Depending on the NF experimental design, we may direct the brain into using one or the other mechanism.

## 5. Summary and conclusions

NF is a procedure that measures brain activity in real time and presents it as feedback to an individual, thus allowing them to self-regulate brain activity with effects on cognitive processes inferred from behaviour. One common argument is that NF studies can reveal how the measured brain activity causes a particular cognitive process. The causal claim often regards the measured brain activity being manipulated as an independent variable, analogous to brain stimulation. However, this causal inference is vulnerable to the argument that there are concurrent changes in other upstream brain activities that cause the changes in the activity generating the feedback. Alternately, NF may causally affect cognition by indirect means. We would argue that researchers should remain open to the idea that the trained brain activity could be part of a causal network that collectively affects cognition rather than being necessarily causally primary. That is, while the NF procedure might target one specific brain region, or one specific connection between two areas, changes in neural activity might ripple across an entire network, thus affecting other regions. The final outcome (cognitive or behavioural change) could depend on activity changes in areas downstream or separate from the NF target. Previous work with functional connectivity NF has indeed shown that post-NF, connectivity patterns beyond the target connection had also changed [112]. This inference may provide a better translation of evidence from NF studies to the rest of neuroscience. The recent advent of multivariate pattern analysis, combined with implicit NF, currently comprises the strongest case for causality [7,24,36,113]; however, future work will have to address this set of questions in depth.

RL and active inference are theoretical models to understand how the goal of optimizing behaviour and learning is achieved through experience. Active inference’s prediction error aligns with RL’s reward prediction error, driving updates to internal models. Active inference’s exploratory drive mirrors RL’s balance between exploration and exploitation. Both frameworks suggest that the brain learns by iteratively refining actions and predictions to achieve desired outcomes.

However, as a model for self-regulation via NF learning RL has fallen short of explaining why some people learn to self-regulate and others do not. RL focuses on maximizing rewards by adjusting actions based on positive or negative outcomes. In the context of self-regulation, this could be applied by considering regional brain activities as actions and the desired brain state as the goal. However, RL might not inherently account for the complex predictive processes and the balance between action, perception and prediction that are central to self-regulation, particularly in explicit NF paradigms. Active inference, on the other hand, explicitly incorporates the idea of minimizing prediction errors and optimizing internal models. It accounts for the interplay between predictions, actions and sensory input, making it well suited to explaining how individuals learn to identify the correct internal actions for self-regulation. The rapid learning observed in self-regulation tasks like NF, where individuals need to identify the correct area for control amidst numerous potential states, can be attributed to a combination of cognitive mechanisms and neural processes, explicitly modelled by active inference in terms of minimization of prediction errors and optimizing internal models. Analyses based on large, pooled datasets across NF studies (e.g. [20,114]) will be critical in better adjudicating the computational processes underlying various NF approaches.

There are also deeper neuroscientific issues that are currently not well understood. Experimental findings in animal studies point to possible brain–behaviour decoupling because of NF training without simultaneous body–behaviour association [115]. Furthermore, the potential extinction of sensory-motor contingency has also been hypothesized with prolonged muscular inactivity in patients with complete paralysis, such as in the vegetative stage and other disorders of consciousness [99]. A phenomenon that may prevent longer term NF effects has been speculated to relate to the ‘rebound’ effect of neural activity resulting in a return to its original activation level after sustained NF training [5].

One limitation of the present approach to NF is related to the lack of bodily and environmental involvement in the NF paradigm. The lack of bodily and contextual settings and conditions that are conducive to brain–body–behaviour modulation during NF training could be the most important limitation of the present approaches to brain self-regulation.

Participants learn to control neural activity using mental strategies, such as emotional scenes, visual imagery, motor imagery and memory recall, without concomitant bodily movements, speech and overt physiological changes because of the restricted environment and the associated physiological artefacts of contemporary brain imaging technology. Furthermore, behavioural and physiological effects of NF are tested before and after training, with very little observation of ongoing changes in these outcomes. Studies are predominantly conducted in static, non-interactionist settings bereft of brain–body–environment interaction. Hence, real-world, socially and ecologically significant interactions are excluded from the contemporary NF approach. Future developments in NF should consider brain–body interactions inspired by embodied-enactive cognition theories. In the proposed approach, the participant is trained in the natural environmental context, which operates in daily life and constrains brain function and behaviour. Embodied cognition (e.g. [116–118]) can be succinctly described as the cognitive processing that includes and extends beyond the agent’s brain in the agent’s ongoing dynamic interactions with the environment. This view begs for innovative techniques to integrate portable and wearable neurophysiological monitoring technology for real-time feedback to train brain and body to act together in a contextually meaningful manner.

## Data Availability

This article has no additional data.
